# Multidisciplinary approach for patients with nephropathic cystinosis: model for care in a rare and chronic renal disease

**DOI:** 10.1590/2175-8239-JBN-2018-0139

**Published:** 2018-11-14

**Authors:** Maria Helena Vaisbich, Carla Aline Fernandes Satiro, Deborah Roz, Debora de Almeida Domingues Nunes, Ana Carola H Lobo Messa, Camila Lanetzki, Juliana Caires de Oliveira Achili Ferreira

**Affiliations:** 1 Universidade de São Paulo, Faculdade de Medicina, Hospital das Clínicas, São Paulo, SP, Brasil.

**Keywords:** Patient Care Team, Cystinosis, Child, Teenager, Adolescent, Equipe de Assistência ao Paciente, Cistinose, Criança, Adolescente

## Abstract

Care for patients with chronic and rare diseases is complex, especially considering the lack of knowledge about the disease, which makes early and precise diagnosis difficult, as well as the need for specific tests, sometimes of high complexity and cost. Added to these factors are difficulties in obtaining adequate treatment when available, in raising patient and family awareness about the disease and treatment compliance. Nephropathic cystinosis is among these diseases. After more than 20 years as a care center for these patients, the authors propose a follow-up protocol, which has been used with improvement in the quality of care and consists of a multidisciplinary approach, including care provided by a physician, nurse, psychologist, nutritionist and social worker. In this paper, each field objectively exposes how to address points that involve the stages of diagnosis and its communication with the patient and their relatives or guardians, covering the particularities of the disease and the treatment, the impact on the lives of patients and families, the approach to psychological and social issues and guidelines on medications and diets. This protocol could be adapted to the follow-up of patients with other rare diseases, including those with renal involvement. This proposal is expected to reach the largest number of professionals involved in the follow-up of these patients, strengthening the bases for the creation of a national protocol, observing the particularities of each case.

## Resumo

A assistência a pacientes com doenças crônicas e raras é complexa, principalmente pela falta de disseminação de conhecimento sobre a doença, o que dificulta o diagnóstico preciso e precoce, além da necessidade da realização de exames específicos, por vezes de alta complexidade e custo. Somam-se a esses fatores dificuldades na obtenção de tratamento adequado quando disponível, na conscientização do paciente e da família sobre a doença e na aderência ao tratamento. A cistinose nefropática está entre essas doenças. Após mais de 20 anos como centro de atendimento a esses pacientes, os autores propõem um protocolo de seguimento, o qual vem sendo empregado com melhora na qualidade da assistência e consiste de uma abordagem multidisciplinar, incluindo, principalmente, atendimento prestado por médico, enfermeiro, psicólogo, nutricionista e assistente social. Neste artigo, cada área expõe de maneira objetiva como abordar pontos que envolvem as etapas do diagnóstico e sua comunicação ao paciente e a seus familiares ou responsáveis, abrangendo as particularidades da doença e do tratamento, o impacto na vida do paciente e de sua família, a abordagem das questões psicológicas e sociais e orientações quanto a medicamentos e dietas. Considera-se que este protocolo poderia ser adaptado ao seguimento de pacientes portadores de outras doenças raras, incluindo aquelas com envolvimento renal. Com essa proposta, espera-se alcançar o maior número de profissionais envolvidos no seguimento desses pacientes, fortalecendo as bases para a criação de um protocolo nacional, observando-se as particularidades de cada caso.

## Introduction

Nephropathic cystinosis (NC) (OMIM 219800) is a rare, autosomal, recessive disease with an incidence of 1: 100.00-200,000 live births,^1^ due to mutations in the CTNS cystinosine-encoding gene, cystine protein-carrier from cell lysosome to cytosol.^2^ With cystinosine dysfunction, there is intralisossomal cystine buildup, which, due to its low solubility, leads to the formation of crystals in different organs, as well as their dysfunction.^1^

In infancy, first, the kidneys are affected with Fanconi's syndrome (FS), and the patient often develops hypothyroidism, gastrointestinal and ocular involvement. Glomerular involvement usually becomes apparent between 2 and 5 years of age, with proteinuria, and results in end-stage renal disease (ESRD) by the age of 10 years. In the second and third decades of life, patients suffer the involvement of other organs, such as the pancreas, skeletal muscles, liver, central nervous system and hypogonadism in males.^3^ Although severe, the disease can be treated with cysteamine, a drug that depletes cysteine stocks, slowing down the progression to ESRD and extrarenal involvement. The earlier the specific treatment starts, the better the patient progresses.^3^

However, even with the ideal treatment, patients may progress to ESRD in the second decade of life and develop extrarenal impairments, especially muscular.^4^^,^ 5 ^,^ 6 Among the factors that contribute to this occurrence; there may be increased oxidative stress, mitochondrial autophagy and increased apoptosis.^7^^,^ 8

In addition, due to the quantity and dosage of the necessary medications, treatment compliance is compromised. One study showed a decreased compliance to cysteamine and reduced motivation to continue with treatment with advancing age.^9^

For 20 years, our service is a reference in the care of patients with cystinosis from 0 to 18 years of age in the country. Currently, 60 patients with NC are followed, four on dialysis and 10 renal transplant recipients. The consultations are weekly in the first month, monthly in the following 6 months and bi-monthly when the metabolic and hydroelectrolyte balance is reached; and patients on conservative treatment in stages 3 and 4 of chronic kidney disease (CKD) of the KDOQI^10^ are always followed monthly. The team responsible for cystinosis evaluates patients on dialysis or transplanted patients every 6 months, and extra consultations are performed as needed.

We developed in our service a multidisciplinary care system for patients with NC, to improve treatment compliance and quality of life of those involved, which is significantly compromised by the impact of clinical manifestations and cystinosis management. The multidisciplinary team is made up of doctors, nurses, nutritionists, social workers and psychologists. The medical consultation is held on a date chosen for the evaluation of each professional, optimizing the discussions of the case and treatment definition.

The following is a breakdown of the main points of approach from each professional.

## Medical care

The doctors talk to the family about the diagnosis and how it was established, explaining the disease to the patient and caregivers, answering questions, following up on the case or making contact with the doctor who will follow the patient.

During the consultations, a proper interview is carried out, verifying acceptance and adverse effects of medications, treatment compliance and complications, physical examination with attention to hydration and nutritional status, bone deformities, photophobia assessment, analysis of test results adequacy of medications.

### Protocol tests

Tests are directed to the evaluation of renal, metabolic and electrolytic function, extrarenal involvement and general patient health. Since this work is done with patients up to 18 years of age, when the protocol is applied to older patients, it should be adapted to extrarenal involvement. Table 1 shows the examinations and the frequency of completion, remembering that they should follow the patient's need and the stage of CKD. Blood and urine samples should be collected in the morning and at the same time. Kidney and urinary tract ultrasonography must be performed annually; and other tests when necessary.

**Table 1:** Protocol of ancillary tests that should be carried out in patients upon diagnosis and follow up

Diagnostic suspicion	Serum tests: sodium, potassium, ionic and total calcium, phosphorus, magnesium, chlorine, venous gases, urea, creatinine, TSH, free T4, lipid profile (total cholesterol and fractions, triglycerides), CBC, fasting glycaemia. Urinalysis: sodium, potassium, chlorine, calcium/creatinine ratio, microalbuminuria/creatinine, creatinine/protein ratio, phosphorus, creatinine, low molecular weight protein (beta 2 macroglobulin, urinary RBP). Urine I + sediment Calculate urinary and plasma anion gap, TPO4/RFG tubular phosphate resorption.
Returns	Serum tests: sodium, potassium, ionic and total calcium, phosphorus, magnesium, chlorine, venous gases, urea, creatinine. Urinalysis: calcium/creatinine ratio, microalbuminuria/creatinine or protein/creatinine ratio. Urine I + sediment
Semester	Serum tests: TSH, free T4, lipid profile (total cholesterol and fractions, triglycerides), fasting glucose, glycated hemoglobin, CBC, PTH, vitamin D, liver enzymes, amylase. Male patients as of 12 years of age: order testosterone.
Annually	Kidney and urinary tract ultrasound

### Treatment protocol

1. Nutrition and water intake: adapted for renal function, laboratory results and estimation of daily diuresis. See section "Nutrition care".
2. Drug treatment includes specific therapy, treatment of FS, the involvement of other organs and adjuvant treatments, such as proteinuria and oxidative stress reduction.

### Fanconi's syndrome treatment

Fanconi's syndrome (FS) due to NC is generally severe, with significant loss of several substances normally reabsorbed by the proximal tubule. Thus, cystinosis treatment begins with the treatment of FS, correcting the hydroelectrolyte and metabolic disorders presented by the patients.

- Alkali Replacement: Sodium bicarbonate or potassium citrate can be used, usually around 10 mEq/kg/day, divided every 8 or 6 hours.
- Potassium replacement: it can be replaced as potassium citrate or as potassium chloride, usually 5-10 mEq/kg of body weight/day, every 8 or 6 hours.
- Sodium can be replaced by sodium bicarbonate, and sodium chloride rarely needs to be added. Sodium bicarbonate or sodium citrate (dose based on the correction of metabolic acidosis) is usually initiated and, according to serum level, the need for dose adjustment is assessed.

In our service we also used a solution called Eisemberg (sodium citrate, potassium citrate and citric acid), with 1 mEq of sodium, 1 mEq of potassium and 2 mEq of base for each 1 ml of the solution. Particularly, the replacement with sodium bicarbonate and potassium chloride has the advantage of replacing sodium, alkali and potassium independently.

- Phosphorus replacement: it can be made as a phosphate solution (15 mg / mL) or in sodium and potassium phosphate tablets, and the tablet contains 250 mg of inorganic phosphorus (in the formulation available from Hospital das Clínicas - FMUSP). The recommended dose is 20-90 mg/kg body weight/day, divided every 8 or 6 hours. It is important to note that higher doses may cause diarrhea and, in the terminal phases of CKD, phosphorus and potassium need to be reduced, so be aware.
- Magnesium Replacement: For patients who need to replenish magnesium, it may be magnesium sulfate (a cheaper formulation but often causes diarrhea), magnesium oxide or magnesium chelate (which has excellent absorption). The replacement should be done according to the serum level and administered every 8 hours. Dose adjustment, if necessary, should be based on serum level.
- Replacement of 25 OH vitamin D: according to needs and serum levels.
- Calcitriol: can be used, but always with monitoring PTH levels, mainly avoiding the occurrence of adynamic bone disease.
- L-carnitine: replace in cases of low plasma levels.^11^

Reference values for total carnitine (µmoles/L) of HC-FMUSP:

- Males: adult 30-70 µmol/L and children 32-62 µmol/L;
- Females: adult 25-58 µmol/L and children 28-59 µmol/L.
- Calcium replacement: if necessary, should be done monitoring ionic calcium. Calcium carbonate is generally used, starting at 400 mg of elemental calcium per day. Care should be taken not to administer calcium with food or with the phosphate formulation, to prevent calcium from reducing the absorption of the phosphate being consumed. Calcium chelate can also be formulated as it has excellent absorption. Generally, hypercalciuria improves with the correction of metabolic acidosis; and there is rarely a need to use hydrochlorothiazide because it worsens hypokalemia, making it necessary to increase potassium replacement. These patients have no tendency to develop nephrocalcinosis or nephrolithiasis, since they present citraturia and bicarbonaturia. However, these events may occur with high doses of calcitriol without monitoring the calciuria.

Non-hormonal anti-inflammatory agents: prostaglandins, especially PGE 2, increases medullary blood flow, inhibits sodium chloride reabsorption in the thick portion of the medullary Henle loop and in the collecting duct, and decreases the expression of aquaporin 2. Thus, inhibition of prostaglandins leads to increased reabsorption of sodium chloride, water and reduction of diuresis. Therefore, the drug may be used, especially in the first 3 years of life, because polyuria is intense and it is sometimes difficult to keep the patient hydrated and to adequately replace the electrolytes. Indomethacin or selective cyclooxygenase 2 inhibitors may be used.^11^^,^ 12 Use of non-hormonal anti-inflammatory drugs should be discontinued as soon as possible to avoid a faster progression to ESRD.

### Specific treatment with a cysteine-depleting drug

This medication, which already has two commercial formulations: immediate release cysteamine (administered every 6 hours) and delayed release cysteamine (administered every 12 hours), is of choice for the treatment of the disease, and responsible for the removal of cystine from lysosomes, attenuating all complications.^3^^-^ 5 The delayed release cysteamine advantage is the dosage, which recommended daily dose can be given every 12 hours. Short-term studies have shown benefits in maintaining the rate of growth and renal function stabilization;^13^ however, further studies are needed to demonstrate the benefits described.

With cysteamine and intralisossomal cystine stocks reduction, there is a prognosis improvement in these patients, delaying progression to ESRD and avoiding or postponing extrarenal involvements.^3^^-^ 5

The immediate cysteamine dosage is 1.3 to 1.9 g/m^2^SC/day, given every 6 hours, with a maximum dose of 500 mg every 6 hours; and the delayed-release cysteamine dosage is 1 g/m^2^ SC/day, given every 12 hours, with a maximum dose of 1 g every 12 hours.

Both cysteamine formulations should always be started with 1/4 of the target dose and gradually increase, according to gastric intolerance, remembering that there is no evidence of the need of dose correction according to renal function.

### How to assess the response to treatment?

The best way to assess treatment response is to measure cystine intraleucocyte content, which should be kept below 1 nmol 1/2 cystine/g protein or, if it is in granulocytes, maintain below 1.9 nmol 1/2 cystine/mg of protein.^14^ We suggest that this test be performed every 4 months, but the KDIGO recommendation is to treat according to body weight when the test is not available.^11^

### Cysteamine side effects

- Unpleasant odor and breath (similar to sulfur), due to the metabolism of cysteamine in sulfur-containing compounds (dimethyl sulfide, metanethiol), which improves with chlorophyll tablets;
- Gastric intolerance, which implies starting treatment at lower doses and increasing it gradually. This effect with delayed-release cysteamine appears to be less severe than immediate-release cysteamine,^11^ in the latter case it improves with omeprazole.^15^ With delayed-release cysteamine there appears to be no need for proton pump inhibitors.^11^
- Effects reported at doses > 1.6 g/m^2^/day of fast-release cysteamine: myalgia, hyperthermia, lethargy, neutropenia, convulsions, allergic rash, proliferative lesions on the skin of the elbows (angioendotheliomatosis), and striae, which may be caused due to copper deficiency, an essential element for the formation of collagen; the absorption of which is compromised by the use of cysteamine.^16^ Patients with FS have increased urinary excretion of copper, indicating compromised transporters involved in proximal tubular reabsorption.^17^ Copper supplementation may prevent cysteamine toxicity or accelerate the reduction of symptoms, when present.^18^ The suggestion is to evaluate the ceruloplasmin serum concentration (copper body biomarker)^19^ and copper; in the case of deficiency and/or increased excretion, copper supplementation must be monitored in serum levels. Based on the recommendation for copper intake according to the age range (Table 2)^19^ and with the serum copper level, if below and adequate intake, it is supplemented until reaching an adequate serum level (serum copper: 80-120 mg/dL, serum ceruloplasmin: 0.22-0.58 g/L, urine copper/creatinine ratio < 50 mg/g).^18^^-^ 19

**Table 2:** Recommended copper intake

Reference Dietary Intake * and maximum copper daily intake		
Age range	Dietary reference	Tolerable upper limit
Adults older than 19 years	900 µg	10 mg
Pregnant women	1000 µg	10 mg; 8 mg se ≤ 18 years
Breastfeeding women	1300 µg	10 mg; 8 mg se ≤ 18 years
Adolescents between 14 and 18 years	890 µg	8 mg
Children between 9 and 13 years	700 µg	5 mg
Children between 4 and 8 years	440 µg	3 mg
Children between 1 and 3 years	340 µg	1 mg
Infants between 7 and 12 months	220 µg**	unavailable
Infants between 0 and 6 months	200 µg**	unavailable

Source: Translated from Harvey et al., 2009.^19^ Notes:

* Daily recommended tolerance;

** Proper intake, recommended daily tolerance not established.

### Treatment of other organs involved

- Ocular involvement: patients initially present with photophobia, which without a specific treatment can progress to amaurosis. The treatment is with cysteamine ophthalmic solution (1 drop in each eye hourly while awake). The formulations approved by the FDA and existing EMA are 0.44% solution and 0.55% gel formulation. In Brazil, a manipulated 0.5% cysteamine solution can be used. The gel formulation enables 4 daily applications (in the morning, at lunch, in the afternoon and at dinnertime) and can be transported refrigerated without freezing.^20^
- Thyroid involvement: Start monitoring at about 2 years of age, measuring TSH and free T4 every 6 months. In the presence of hypothyroidism, initiate thyroid hormone replacement.
- Pancreatic involvement: By age 5, patients may present with glucose intolerance, but diabetes mellitus usually develops in the second or third decade of life. Monitor fasting glycaemia and glycated hemoglobin annually from 5 years of age.
- Muscular impairment: one of the most serious late complications that can determine respiratory and swallowing difficulty is muscle weakness with generalized atrophy, beginning distally. Studies have shown that the use of cysteamine slows this effect.^3^

The other involvements must be treated according to their manifestations.

### Proteinuria treatment

Some authors have advocated the use of an angiotensin converting enzyme inhibitor or angiotensin receptor blocker as an antiproteinuric agent; however, these patients are prone to hypovolemia; therefore, there is a risk of worsening of glomerular function.^21^ In our experience, the use of these drugs does not bring benefits, as serum creatinine is frequently increased.

### Oxidative stress reduction

Since patients with lysosomal deposition disease, particularly cystinosis, present increased oxidative stress, the use of an antioxidant drug could bring benefits. As demonstrated in a study carried out at the Children's Institute, N-acetylcysteine was beneficial in decreasing the marker of oxidative stress (TBARS-reactive substances) and preserving glomerular renal function in patients with cystinosis.^22^ We propose that this drug is used as a coadjuvant in the treatment of these patients.

Table 3 shows an instrument used for guidance on medications, which is filled out together with the caregiver and adapted to the family's routine, considering the following items:

**Table 3:** Template of a spreadsheet to be given to the patient and his caregiver, with instructions as to medication times, doses and observations

Medication		Vitamin D (200 UI/drops)	Phosphate solution 15 mg/mL	Potassium chloride 10%	Sodium bicarbonate 10%	Fast-release cysteamine (every 6 hours) 150 mg or slow-release cysteamine (every 12 hours)	Calcitriol 0,25 mcg	Levothyroxine 25 mcg
Dose		5 drops	10 mL	15 mL	20 mL	2 tablets	1 tablet	1 tablet
Time of medication	6h	x	x	x				x
	7h				x	x	x	
	12h		x	x				
	13h				x	x		
	18h		x	x				
	19h				x	x		
	00h		x	x	x	x		

Source: Instituto da Criança HCFMUSP, 2018.

- Electrolyte and alkali replacement should be fractionated in 3 to 6 doses per day;
- do not offer all the replacements at the same time, as it can cause vomiting;
- Medication schedules should consider particularities such as the need for fasting or relation to food offered simultaneously;
- Medication must be offered by one or at most two caregivers;
- In the case of adolescents, one must encourage autonomy in relation to the administration of medicines, but adult supervision is essential.

### Cystinosis and dialysis

The fact that patients with cystinosis are polyuric and have significant diuresis all the way to advanced stages of CKD make them free of hypervolemia issues; thus, the best method for dialysis is peritoneal (PD), in which residual renal function is better preserved.^23^ When it is not possible to perform PD and the patient undergoes hemodialysis, it is important that there are no major changes in volume, that may promote loss of residual renal function and decrease of diuresis.

### Cystinosis and renal transplantation

Renal transplantation (TxR) is the best form of RRT in NC, because, in addition to the disease not recurring in the graft, the prognosis is excellent.^24^

A study comparing patients transplanted with NC and transplanted by other causes, matched by age, date of TxR, type of donor and site of TxR, showed greater graft survival in NC patients, and there was no difference in the rate of diabetes mellitus between the two groups with the use of corticosteroids and tacrolimus. Therefore, the recommendation is to maintain the standard immunosuppressive regimen and monitor it.^24^

As patients with cystinosis often present significant residual diuresis, the possibility of preemptive transplantation is relevant.

In our experience, the graft prognosis is excellent, but without the specific treatment, the patients present a high percentage of extrarenal involvement.

## Nursing care

The success of treating children with cystinosis and improving their quality of life depends directly on family compliance. The family needs to be clear about the disease, the possible complications and their importance throughout the process. The family must receive care, as well as the child, and know that their actions should go beyond the correct administration of medications.

The nurse has a differentiated action in outpatient procedures and hospitalization of the cystinotic patients:

- outpatient clinic: anthropometric measurements, vital signs measurement, vaccination status check, collection of laboratory tests, follow-up of family compliance, check for patient's and caregiver's understanding of the disease and treatment, application of quality-of-life questionnaires;
- hospitalization: detailed interview, especially on the reason for hospitalization, treatment status, family history, vaccination status; physical examination with attention to the skin (turgor, temperature, dryness, lesions), mucous membranes, edema, respiratory frequency and sounds, blood pressure, heart rate and rhythm, behavioral changes, paresthesia, limb weakness and tremors.

Nurses must also work with the family on the issue of water intake and medication administration, raising awareness concerning problems stemming from poor compliance.

Regarding the guidelines for specific medication (drug depleting intralisossomal cystine stores); the nurse must know the two commercial types available and make the guidelines according to the manufacturer's recommendations.

- Delayed-release cysteamine: can be ingested with food, including milk, which improves digestive tolerance. Concomitant ingestion of acidic foods should be avoided.
- Immediate-release cysteamine: avoid eating 2 hours before until 30 minutes after; avoid dairy products within 1 hour of administration. The capsule can be ingested intact with water and the granules can be mixed with citrus juices or fruit jelly.

Nurses also act directly in the care of patients who progress to dialysis, preferably peritoneal dialysis (PD), which can be performed at home with automated machines, contributing mainly to family education for therapy.

## Nutritional care

NC nutritional management is done according to food acceptance, nutritional status and renal disease stage.

The initial disease stage (stage 1 CKD) is characterized by hydroelectrolytic and metabolic disorders by FS. According to age and nutritional status, in this stage the orientation is normal to hypercaloric diet, normo-protein and adequate in micronutrients, according to the Dietary Reference Intakes (DRIs).^25^

Patients are generally inept due to the influence of polydipsia, polypharmacy and gastric intolerance, characteristics of the disease. They are avid for salty foods and have low acceptance for sweets, which makes it difficult to use dietary supplements, since most of them have a sweet taste. Thus, we increase caloric density by adding unsaturated fats, complex carbohydrates and proteins in daily preparations. Another strategy is the use of spicy and sour flavor foods, such patients prefer foodstuff such as lemon and pepper.

When oral feeding does not meet the nutritional need of the patient, enteral therapy is indicated. If the gastrostomy is performed, we suggest to mantain during the first decade of life due to anorexia.^26^ Nutritional therapy should be individualized, with schedules, choice of enteral diet, volume and infusion time according to patient tolerance. The caloric and protein requirement is calculated according to the DRIs.

Growth deficiency in NC patients is usually higher compared to other nephropathies, so nutritional treatment aims at optimizing weight gain.^27^ However, it often requires follow-up with an endocrinologist to define the need for growth hormone replacement therapy.

Water intake is free, except when diuresis is reduced. It is important to avoid giving water immediately before, during and immediately after meals to minimize food refusal and avoid vomiting episodes.

Feeding is assessed using quantitative and qualitative instruments, such as the food frequency questionnaire, food record, 24-hour food recall or usual food recall. It is also important to evaluate gastrointestinal issues, since patients with NC tend to present abdominal pain and distension, nausea, vomiting, intestinal constipation and/or diarrhea.^28^

Thus, it is fundamental to understand the patient's routine, medication and sleep schedules, water intake and food preferences to establish an individualized plan.

The anthropometric evaluation should be complete, with standardized measurements, and made with calibrated equipment. The measures used are weight, height, head circumference, skin folds and circumferences.^29^ For weight measurement, the child should be naked and barefoot and the supply of liquids immediately prior to weighing should be avoided. In order to measure height, the child should be barefoot and unadorned in the head, it is possible to use the horizontal or wall stadiometer, according to patient age.^29^ For patients under one meter, the horizontal stadiometer is used, even for those older than 2 years, because of the bone deformity, this methodology allows a more reliable evaluation. With the weight and stature data, it is possible to evaluate the indices: Weight for Height, Height for Age, Weight for Age and Body Mass Index (BMI) for age, with percentile or z-score evaluation according to World Health Organization recomendations.^30^ The calculation is made using the Anthro and Anthro Plus software.

Skin folds and circumferences assessments are fundamental for body composition assessment. The main measures used are: arm circumference and triceps skinfold, using the Frisancho reference.^31^

The disease progresses to ESRD, so it is important that the nutritionist is integrated with the multidisciplinary team to identify the ideal timing of change in dietary management.

The adequacy of protein consumption should be started from the diagnosis of CKD 2. In addition to the protein adjustment, the evaluation of the total caloric supply is indispensable, so that the ratio of protein to non-protein calories is adequate, avoiding catabolism and providing a better-balanced nitrogen. At the Instituto da Criança, the caloric need calculation is according to DRIs, and the protein requirement is defined according to the total energy value, which considers gender and age (Tables 4 and 5). In this phase, carbohydrate or lipid modules are often used for a better supply of non-protein calories.

**Table 4:** Protein and caloric supply for patients with Chronic Kidney Disease under conservative treatment

Conservative Treatment				
Stage	1-3 years	4-6 years	7-10 years	11-18 years
I and II	20% VET	Adequate for age (with no excesses)	Adequate for age (with no excesses)	Adequate for age (with no excesses)
III	15 to 20% VET	15 to 20% VET	20 to 25% VET	20 to 25% VET
IV and V	10 to 15% VET	10 to 15% VET	15 to 20 % VET	15 to 20% VET

Source: Instituto da Criança HCFMUSP, 2018. Note: VET = total energy value.

**Table 5:** Caloric and protein supply protocol for Chronic Kidney Disease patients under dialysis

Hemodialisis				
Stage	1-3 years	4-6 years	7-10 years	11-18 years
IV and V	20%	20 a 25%	25%	25%
Peritoneal Dialysis				
Stage	1-3 years	4-6 years	7-10 years	11-18 years
IV and V	15% VET (in average at 3.5g/kg/day)	15 to 20% VET	20 to 25% VET	25% VET

Patients with NC have electrolyte losses and receive replacement even after the CKD diagnosis, so most of them have no indication of a diet low in potassium and phosphorus and low-sodium unless they are in an advanced stage of CKD, and these parameters are altered.

Sodium restriction, commonly indicated by healthcare professionals working with CKD, may worsen the renal function of these patients if there is still significant sodium loss. The same goes for the other electrolytes.

## Psychological support

With advances in treatments for chronic diseases, healthcare professionals are increasingly faced with the care of children with serious and long-term pathologies. NC is a paradigmatic example of this new reality.

The diagnosis of a chronic disease has an impact. It is a traumatic contingency of life that affects preexisting expectations.

The desire to have a child implies a mechanism of idealization of the child. Already in the gestation, there is an imaginary construction of a place for this child, to the extent that he can be or accomplish everything in which we think we have failed. Sigmund Freud used the term: "His majesty the baby" to express this privileged place given to the baby by the parents.^32^

The sickness of a child causes a concussion in these fantasies. The internal conflicts of this painful experience trigger defense mechanisms in the parents, who may try, for example, to attribute to the partner the blame for the disease transmission, and so often, the couple separates. Because it is a genetic disease, NC has made parents guilty of having failed in their ability to raise a healthy child.

It can also shake the financial conditions of the family, as the mother may need to stop working to care for the child. The relentless routine of medication schedules, especially at the beginning, makes the mother's life revolve only around her child, and she ends up giving up on her personal projects, in addition to having her attention charged by her husband and the other children.

The baby who does not grow and does not gain weight, the pilgrimage to doctors and the difficulties in obtaining a diagnosis have repercussions in the construction of bond between mother and baby, with risks to the good psychic organization of the child.

In adapting to a chronic illness, the family can exert anxious overprotection over the child, rejection, denial, omnipotence or, then, tolerant and realistic acceptance.^33^ Children may present, in the course of time, a position of passive dependence, rebellion, and opposition to medical advice or, at best, a realistic acceptance of their condition.^34^

The ICr multidisciplinary team began to perform group meetings with parents of cystinotic children on outpatient days. In these groups, parents talk about their experiences and anxieties about their children's illness. This device proved to be a privileged instrument for the rapprochement between parents and staff and between the parents themselves, creating a network of complicity and partnership in the search of resolution for the impasses related to the disease. In addition to the groups, the psychologist particularly responds to the spontaneous demands of patients and/or families or when requested by the multidisciplinary team.

The care given to these families has taught us that the acceptance of their anxieties and the trust that is established with the team are essential elements for the long and arduous treatment.

## Social Service Support

The work of Social Services is centered in the identification of social, economic and structural problems that interfere with treatment access and compliance. The disease is the cause of limitations and changes of impact in the lives of patients and their relatives. Situations that manifest feelings of pain, suffering, loss and fragility.^35^

During the social interview, such data as: family structure, socioeconomic evaluation, housing conditions and social resources, provide elements that enable understanding the initial conditions for coping with the disease and to develop strategies to support these individuals.

As our institution has been considered a reference center for the treatment of cystinosis in infancy, the family usually arrives distressed to the hospital, upon diagnosis confirmation and expectations concerning the future life. In this new situation, the family needs to reorganize and adapt.

We observed that patients have difficulties attending school, for reasons such as frequent consultations, hospitalizations, examinations, some limitation or even insecurity of the family in allowing them to live with other children and the care offered on the spot.

In Brazil, we guide and refer families that are in a situation of social vulnerability to resources/assistance benefits as support for treatment, but we find difficulties in relation to the effectiveness of public policies. We work with government support, such as free public transportation, lodging, food, financial assistance for children with disabilities, judicialization for the purchase of high-cost medicines, and for the guarantee of the rights guaranteed in the policies of Attention and Protection to Children and Adolescent - Federal Constitution (Brasília, 1988); Statute of the Child and Adolescent (Law no. 8.069/1990) and the Unified Health System itself (Law nº 8.080/1990).

In patients under risk and having exhausted all possible approaches and interventions with families related to treatment compliance, we refer the patient to judicial follow-up.

## Discussion

In Brazil, in addition to the lack of precise epidemiological data, the disease is probably underdiagnosed. There are few studies on this subject, among which we highlight a multicenter study conducted between 1999 and 2008, with the objective of making a demographic assessment of cystinosis, especially on its renal involvement, extrarenal manifestations and its progression with specific treatment.^5^ In this study, 102 cases were identified.

Our current data show about 140 patients with cystinosis in Brazil, but it is only an estimate based on internal registry, including patients in our service, cases of patients who came to diagnosis and returned to the service of origin and cases reported by colleagues.

At present, there is still a shortage of trained professionals to diagnose and treat patients with cystinosis.

Due to the characteristics of this chronic and systemic disease, in which there is a daily need for many medications, special attention should be given to patients and caregivers.

In this study, the authors propose a multidisciplinary approach in the care of these patients and report their experience proposing objective tools for their care. This approach includes medical, nursing, nutritionist, social worker and psychologist care, thus obtaining a comprehensive evaluation of the patient and his family.

Regarding medical care, the consultation includes several items and needs at least 40 minutes. In most cases, great difficulty is observed, especially in relation to the understanding of the disease and the dosage of medications. After completion of the consultation with each specialist, the conduct should be reviewed and the patient and caregiver should be given time to ask questions.

## Conclusion

The care of patients with severe and chronic diseases requires a multidisciplinary approach, which occurs in cystinosis, a serious and progressive systemic disease that requires a series of care. Our experience with this type of approach has demonstrated improvements in compliance to treatment and quality of life, with special emphasis on instruments such as parenting, home visits, medication-related specific instructions and team meetings as differential factors for treatment excellence.
